# Cancer *Evo–Dev*: A Theory of Inflammation-Induced Oncogenesis 

**DOI:** 10.3389/fimmu.2021.768098

**Published:** 2021-11-22

**Authors:** Wenbin Liu, Yang Deng, Zishuai Li, Yifan Chen, Xiaoqiong Zhu, Xiaojie Tan, Guangwen Cao

**Affiliations:** ^1^ Department of Epidemiology, Second Military Medical University, Shanghai, China; ^2^ School of Public Health, Shandong First Medical University & Shandong Academy of Medical Sciences, Tai’an, China; ^3^ Department of Nutrition, School of Public Health, Anhui Medical University, Hefei, China

**Keywords:** cancer, evolution, inflammation, mutation, viral infection

## Abstract

Chronic inflammation is a prerequisite for the development of cancers. Here, we present the framework of a novel theory termed as Cancer Evolution-Development (*Cancer Evo-Dev*) based on the current understanding of inflammation-related carcinogenesis, especially hepatocarcinogenesis induced by chronic infection with hepatitis B virus. The interaction between genetic predispositions and environmental exposures, such as viral infection, maintains chronic non-resolving inflammation. Pollution, metabolic syndrome, physical inactivity, ageing, and adverse psychosocial exposure also increase the risk of cancer *via* inducing chronic low-grade smoldering inflammation. Under the microenvironment of non-resolving inflammation, pro-inflammatory factors facilitate the generation of somatic mutations and viral mutations by inducing the imbalance between the mutagenic forces such as cytidine deaminases and mutation-correcting forces including uracil–DNA glycosylase. Most cells with somatic mutations and mutated viruses are eliminated in survival competition. Only a small percentage of mutated cells survive, adapt to the hostile environment, retro-differentiate, and function as cancer-initiating cells *via* altering signaling pathways. These cancer-initiating cells acquire stem-ness, reprogram metabolic patterns, and affect the microenvironment. The carcinogenic process follows the law of “mutation-selection-adaptation”. Chronic physical activity reduces the levels of inflammation *via* upregulating the activity and numbers of NK cells and lymphocytes and lengthening leukocyte telomere; downregulating proinflammatory cytokines including interleukin-6 and senescent lymphocytes especially in aged population. Anti-inflammation medication reduces the occurrence and recurrence of cancers. Targeting cancer stemness signaling pathways might lead to cancer eradication. *Cancer Evo-Dev* not only helps understand the mechanisms by which inflammation promotes the development of cancers, but also lays the foundation for effective prophylaxis and targeted therapy of various cancers.

## 1 Introduction

Non-resolving inflammation, which is frequently related to chronic infection, chronic pollution, metabolic syndrome, ageing, and physical inactivity, is a prerequisite for the development of most cancers. Many efforts have been devoted to revealing the mechanisms by which inflammation promotes carcinogenesis. For instance, since the association between hepatitis B virus (HBV) and hepatocellular carcinoma (HCC) was determined in 1975, hepatitis C virus (HCV), food contaminants, tobacco smoking, and environmental toxins are identified to cause HCC *via* inducing chronic inflammation (1, 2). Most of these studies provide segmental and fragmental evidence, while only a few present a theoretical hypothesis that deciphers the fundamental laws involved in inflammation-induced carcinogenesis. In past decades, attempts have been made to consecutively investigate carcinogenesis from an evolutionary point of view (3). In 1976, Peter Nowell first proposed that most neoplasms originate from a single cell and Darwinian natural selection occurs during the clonal expansion (4). In 2006, Lauren Merlo described the selection and accumulation of mutation during the process of cancer development. However, only a limited number of gene mutations and related signaling pathways were discussed (5). Widespread application of new generation sequencing promotes the understanding of the landscape of whole cancer genome. Michael Stratton reported that the occurrence of somatic mutations is semi-random, whereas the accumulation of somatic mutations is directed during oncogenesis (6). In 2013, some mutation patterns in the cancer genome were linked with a group of specific mutagenic factors, such as age, inflammation, smoke, and ultraviolet radiation. These mutation patterns were termed as mutation signatures (7). The inflammation-induced mutation signature is dominant in most cancers (8). In 2014, the law in the co-evolution of HBV and cancer cells during chronic inflammation was elucidated with epidemiological and experimental evidence (9). In 2019, we identified the correlations among the genetic polymorphisms, HBV infection-induced inflammation, and imbalance between the mutagenic forces and mutation-correcting forces (10). We also identified a “dead-end evolution” of HBV during chronic infection (11). Here, we present the theory of Cancer Evolution-Development (*Cancer Evo-Dev*) originally *via* summarizing the evidence from our studies on HBV-induced carcinogenesis and then other inflammation-related carcinogenesis. The theory of *Cancer Evo-Dev* not only enriches the understanding of the inherent law of carcinogenesis but also promotes the development of specific prophylaxis and targeted therapy.

## 2 The Framework and Rationale of *Cancer Evo-Dev*

**Figure 1:**
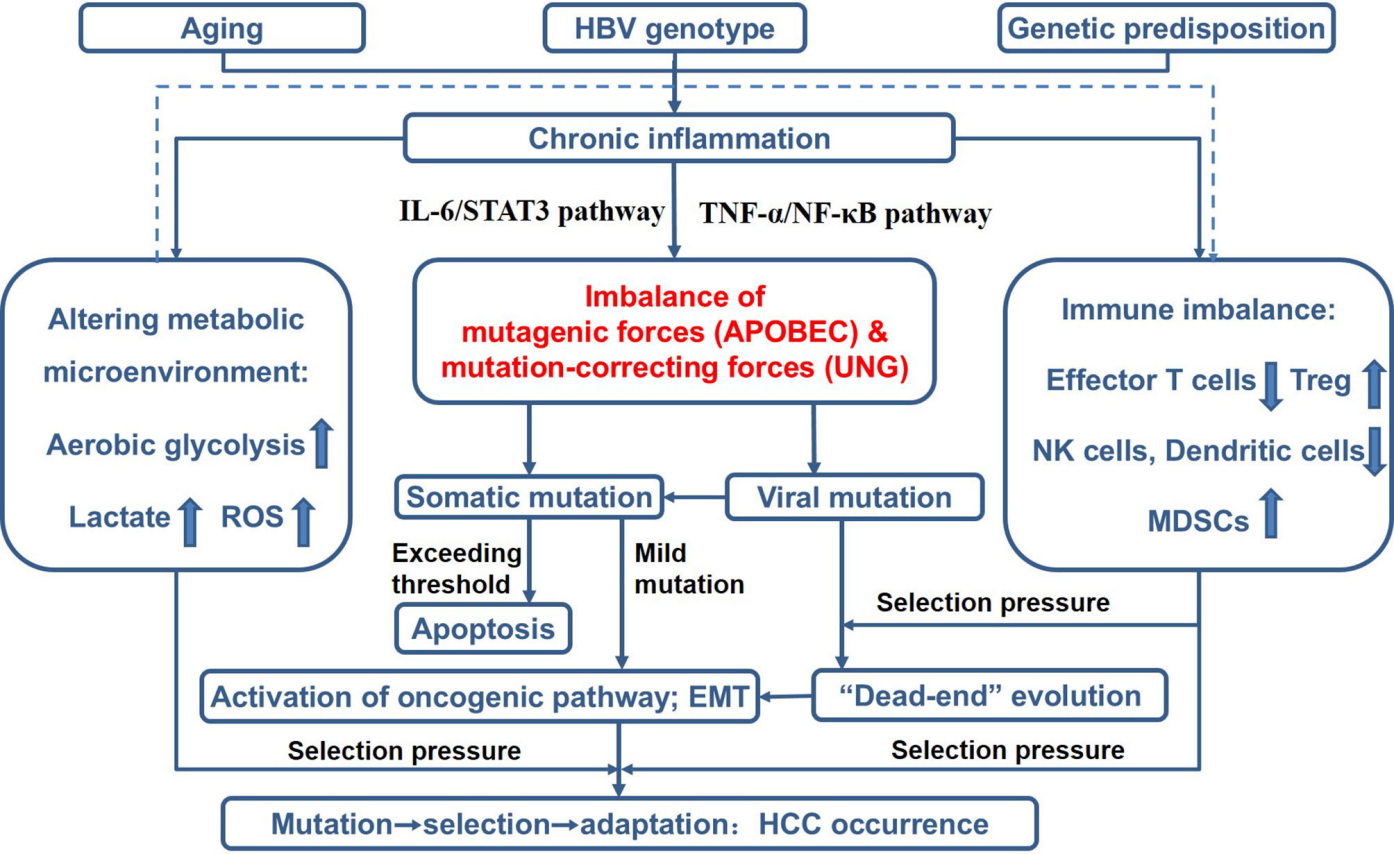
Theoretical framework of *Cancer Evo-Dev*, as exampled by HBV-induced hepatocarcinogenesis. ROS, reactive oxygen species; Treg, regulatory T cells; NK cells, natural killer cells; MDSCs, myeloid-derived suppressor cells; EMT, epithelial-mesenchymal transition.

By summarizing evidence from a variety of perspectives, we gradually proposed and modified the theory of *Cancer Evo-Dev* (12). The framework of this theory is as follows (**Figure 1**): Aging, environmental exposures, and genetic predisposition contribute to the activation and maintenance of non-resolving inflammation that functions as the microenvironment for cancer evolution. Non-resolving inflammation induces dramatic metabolic changes and immune imbalance that served as selection pressure. Activated inflammatory signaling pathways can *trans-*activate the expression of mutation-promoting forces including the human apolipoprotein B mRNA-editing enzyme catalytic polypeptides (APOBECs) family cytidine deaminases and *trans-*inactivate the expression of mutation-correction forces including uracil–DNA glycosylase (UNG), thus promoting viral and somatic mutations. Viral mutants facilitate the malignant transformation of normal cells. Most mutant cells are eliminated under the selective pressure of the pro-inflammatory microenvironment, while a small proportion of mutated cells survive. These survived mutant clones are then retro-differentiated into tumor-initiating cells *via* altering the original cell signal patterns, promoting epithelial-mesenchymal transition (EMT), and reprogramming the metabolic patterns. In turn, the mutant cells and viruses also affect the inflammatory microenvironment. Some established cancer markers, such as α-fetoprotein (AFP) and carcinoembryonic antigen (CEA), are usually expressed at the embryonic stage during individual development, silenced after birth, and re-expressed in cancer patients. These pieces of evidence imply that the process of cancer evolution and development can be characterized as “backward evolution” and “retro-differentiation”.

### 2.1 Chronic Inflammation Is Indispensable for *Cancer Evo-Dev*

As a defense mechanism responding to exogenous infection and injury, acute inflammation is beneficial to humans. However, chronic inflammation, also termed non-resolving inflammation is essential for carcinogenesis. Low-level chronic inflammation is also a hall marker of aging, the dominant cause of most cancers. Cancer evolution is based on two conditions: the continuous acquisition of somatic or viral mutations and natural selection acting on the resultant phenotypic diversity. Chronic inflammation fulfills these two conditions by inducing mutagenic factors such as APOBECs and providing selection pressure.

#### 2.1.1 Chronic Inflammation Facilitates the Generation of Mutations Through Up-Regulating APOBECs

The APOBECs are powerful endogenous mutagenic factors that can catalyze cytidine deamination to create cytosine-to-uracil (C>U) and guanosine-to-adenosine (G>A) transitions. The members of APOBECs family, including APOBEC3 and activation-induced cytidine deaminase (AID), play important roles in the innate immune system (9). APOBEC3s contribute to the elimination of viruses through increasing the viral mutation load to a level that exceeds the threshold of viruses’ replication. APOBEC3s can also induce somatic mutations in host genome. Three mechanisms prevent the APOBEC3s-induced somatic mutation from exceeding the ability of DNA repair. First, APOBEC3s especially APOBEC3A are rarely expressed in normal tissues. Short-term activation of APOBEC3s is beneficial for eliminating pathogens. Second, the cytidine deaminase activity of APOBEC3s is applied almost exclusively to single-stranded nucleotides, in which mutagenesis is 200–300 times more efficient than it is in double-stranded DNA. Third, the uracil-induced mutagenesis of APOBEC3s is counteracted by UNG that plays an important role in the base-excision repair (BER) mechanism (9, 13). However, under the microenvironment of chronic inflammation, signaling pathways including interleukin 6 (IL-6)/signal transducer and activator of transcription 3 (STAT3) pathway and tumor necrosis factor α (TNF-α)/nuclear factor kappa B (NF-κB) pathway are activated, which lead to the long-term upregulation of APOBEC3s (9). Besides, the DNA repair function was also inhibited by inflammatory factors. IL-6 can decrease the expression of UNG while increasing the expression of APOBEC3B. The functional polymorphisms located in the APOBEC3B promoter (rs2267401-G) and UNG enhancer (rs3890995-C) predispose the IL-6 induced APOBEC3B-UNG imbalance and increase the risk of HCC (10). The mitochondrial (UNG1) and nuclear (UNG2) forms of human uracil-DNA glycosylase, both of which are encoded by the UNG gene, are generated by alternative transcription starts, making use of an exon specific for the N-terminal end of the nuclear form, and alternative splicing. UNG2 (open reading frame (ORF) 313 amino acid residues) differs from UNG1 (ORF 304 amino acid residues) in the 44 amino acids of the N-terminal sequence, which is not necessary for catalytic activity. The catalytic domain is present in both UNG1 and UNG2 (14). The transcription of UNG1 and UNG2 should be regulated by their enhancer. As the UNG enhancer activity can be significantly inhibited by IL-6, the expression of UNG1 is expected to be suppressed in the presence of IL-6. Thus, the mutagenic force serving as antiviral immunity is prone to damaging the human genome under the microenvironment of chronic inflammation. The molecules contributing to the imbalance between the mutagenic forces and mutation-correcting forces are varied in different tissue types. A study of colorectal cancer (CRC) demonstrated that APOBEC3G facilitates hepatic metastasis of CRC in mouse models (15). Mutations in genes involved in the mismatch repair (MMR) pathway led to the development of Lynch syndrome and increase CRC susceptibility (16). Thus, non-resolving inflammation may promote carcinogenesis *via* up-regulating APOBEC3A, 3B, and 3G and down-regulating UNG and MMR, leading to the imbalance between the mutation-promoting forces and the mutation-correcting forces.

**Figure 2:**
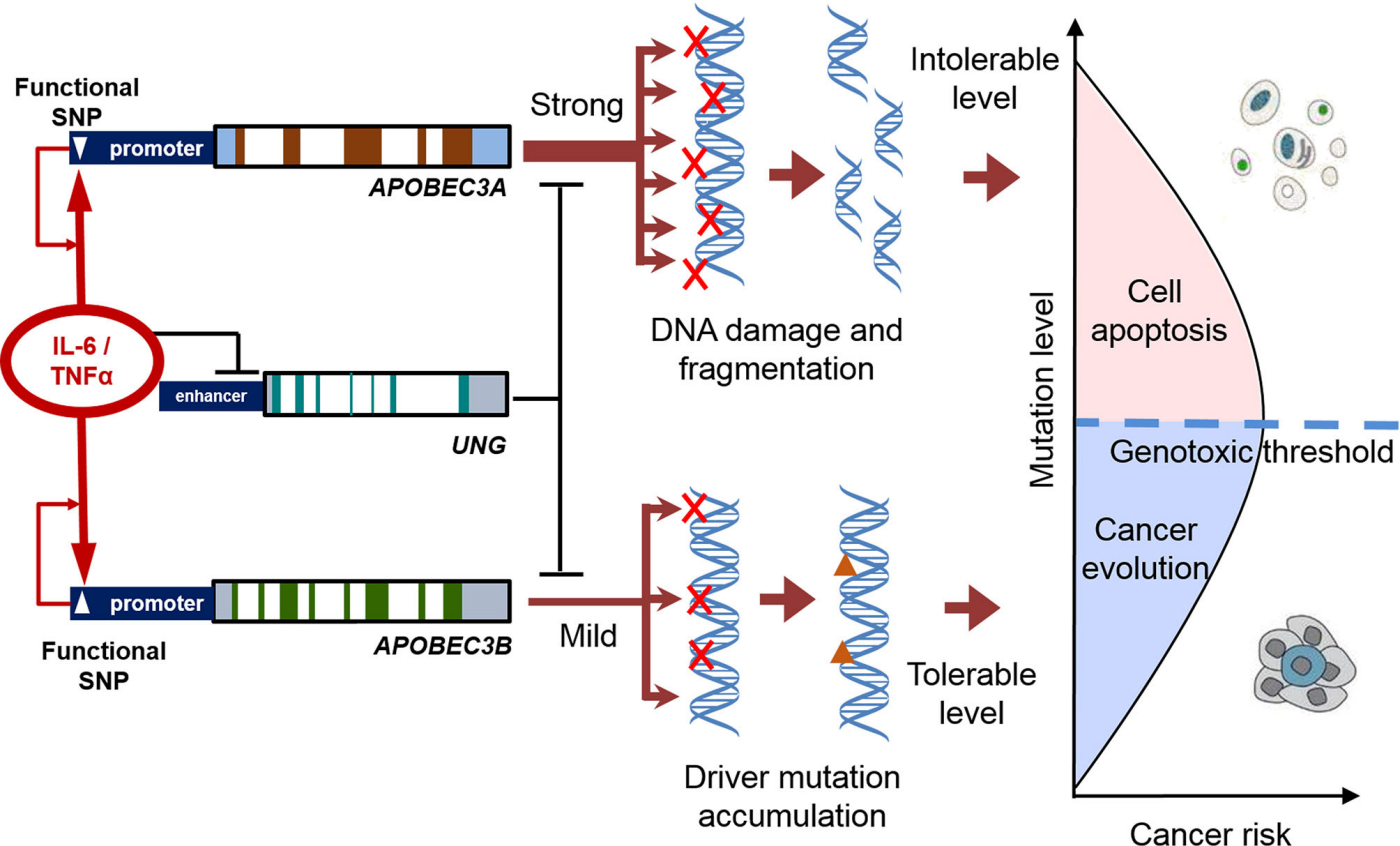
The balance between mutagenic activity and tolerance of cells to genotoxicity in the process of cancer evolution. Inflammatory factors increase the expression of APOBECs and decreased the expression of UNG. The functional polymorphisms enhance the APOBEC-UNG imbalance and promote the generation of somatic mutation (and viral mutation). A tolerable increase in somatic mutations can improve the diversity of mutant cells. If the mutations exceed the tolerable limit, affected cells undergo apoptosis, instead of developing into cancer-initiating cells.

The balance between mutagenic activity and the tolerance of cells to genotoxicity is also important for cancer evolution. A tolerable increase in somatic mutations can improve the diversity of mutant cells. However, if the mutations exceed the tolerable limit and affect the basic function of cells, mutant cells undergo apoptosis, instead of developing into cancer-initiating cells (**Figure 2**). The mutagenic efficiency of APOBEC3A is much higher than that of APOBEC3B. The functional polymorphisms that increased the expression of APOBEC3A significantly decreased the risk of renal cell carcinoma (RCC). Ectopic expression of APOBEC3A increases the apoptosis of RCC cells (17). Another study also demonstrates that the homozygotes allele promoting APOBEC3A expression improves the prognosis in bladder cancer, while the heterozygotes genotype is significantly associated with a poor prognosis (18). Although the APOBEC3A induced mutations are common in cancers, APOBEC3A is often expressed with extremely low abundance. The inflammatory pathways activated by viral infection can only transiently up-regulate APOBEC3A (19). The above genetic polymorphisms may decrease the risk of cancers through breaking the tightly controlled regulation of APOBEC3A.

#### 2.1.2 Maintenance of Chronic Inflammation

The interaction between environmental exposure and genetic predispositions contributes to the maintenance of chronic inflammation. This is particularly evident in maintaining chronic infection of HBV. HBV can be classified into eight genotypes (A to H). The predominant one in mainland China is genotype C (68.3%), followed by genotype B (25.5%) (20). Genotype B HBV is prone to causing acute infection, whereas genotype C HBV is associated with chronic infection and contributes predominantly to the development of HCC (21). The single nucleotide polymorphisms (SNPs) in the loci encoding human leukocyte antigen class II (HLA-II), NF-κB, and STAT3 are significantly associated with the risk of HBV-induced HCC (22–26). Interestingly, the allele frequencies of SNPs affecting the expression of HLA-DP, HLA-DR, and HLA-DQ differ greatly among human races. The polymorphic genotypes that significantly increase the risks of chronic inflammation, HCC, and the generation of high-risk HBV mutations are more frequent in the Han Chinese than in European populations (27). These data suggest that the Han Chinese is inherently more apt to progressing into chronic infection once exposed to HBV infection than Europeans. This might be partly responsible for the fact that chronic HBV infection, HBV-induced liver cirrhosis, and HBV-HCC are more frequent in Chinese than in European populations.

The frequency of APOBEC3B deletion also differs greatly among human races. The frequency of APOBEC3B deletion is 6.49% in European population, 0.9% in African population, and 36.86% in East Asian population (28). APOBEC3B deletion creates a chimera with the APOBEC3A coding region and APOBEC3B 3’UTR. The mutagenic effect of APOBEC3A-B is stronger than APOBEC3B (29, 30). APOBEC3B deletion has been demonstrated to increase the risks of developing non-small cell lung cancer (NSCLC), oral squamous cell carcinoma, and HCC in Chinese population (31–33). The associations of APOBEC3B deletion with the risk of cancers in European population are controversial. It has been shown that APOBEC3B deletion increases the risks of breast cancer and bladder cancer in European, American, and Japanese population (18). However, no significant association between cancer risks and APOBEC3B deletion was observed in European population (34). More population-based studies are needed to consolidate the association.

As one of the most important risk factors of cancer, ageing also plays an important role in the maintenance of low-level chronic inflammation. In cells with senescence, the widespread epigenetic alteration dramatically increases the secretion of proinflammatory cytokines and chemokines (35). The secretome of aging cells is termed as senescence-associated secretory phenotype (SASP) including IL-1α, IL-6, IL-8, IL-10, and granulocyte-macrophage colony-stimulating factor (GM-CSF) (36). SASP factors activate oncogenic signaling pathways such as the Wnt pathway and facilitate the proliferation and invasion of cancer cells (37, 38). The accumulation of SASP cells also appears to induce a positive feedback loop within their microenvironment through inducing IL-6 and plasminogen activator inhibitor-1 (PAI1) (39, 40). Thus, aging can induce chronic inflammation.

Pollution, metabolic syndrome, physical inactivity, and adverse psychosocial exposure also promote the development of cancers *via* inducing chronic low-grade inflammation (41–49). Environmental and psychosocial factors and nutrition contribute mostly, either with a causative or a promotional role to increased low-grade smoldering chronic inflammation, especially in aged population (44). The development of metabolic syndrome is attributed to the chronic low-grade inflammation that occurs in metabolic tissues including the liver (45). People with glucose intolerance, sedentary behavior, obesity, and lower physical activity levels have higher levels of circulating inflammatory factors that have been associated with increased risks of different cancer types (liver, endometrial, colorectal, esophageal, renal, pancreatic, gastric cardia, ovarian, meningioma, postmenopausal breast, multiple myeloma, gallbladder, thyroid, and lung) (46–48). Inflammation can be classified as “good” and “bad” inflammation. A smoldering chronic inflammation, one kind of “bad” inflammation (49), contributes greatly to cancer evo-dev.

#### 2.1.3 Inflammation Provides Selection Pressure

Both aging- and infection-induced chronic inflammation regulate the selection pressure of mutated cells *via* altering immune orientation in the proinflammatory microenvironment. Aging primarily affects immunity through a reduction of primary lymphopoiesis. The quality and the number of lymphoid progenitor cells reduce with age and the cellular immune compartment becomes skewed toward a myeloid lineage, thus leading to immunosenescence. Immunosenescence, a hallmark of aging, is characterized by the dysfunction of effector immune cells including effector T cells, natural killer (NK) cells, macrophages, and dendritic cells. Meanwhile, the levels of immunosuppressive myeloid-derived suppressor cells (MDSCs) and regulatory T (Treg) cells are upregulated by aging (50, 51). In addition, aging perturbs the inflammatory state by increasing secretion of pro-inflammatory cytokines including IL-1, TNFα, IL-6, and C reactive protein (CRP) (49). Thus, aging proved a weaker selective pressure for malignant cells. Another hallmark, SASP, also induces dramatic metabolic changes such as mitochondrial dysfunction, hydrogen peroxide production, and a switch towards aerobic glycolysis. These changes lead to increased production and secretion of lactate and reactive oxygen species (ROS) (52). As discussed above, the expression of mitochondrial UNG1 should be suppressed in the inflammatory microenvironment with IL-6. Inactivation of the UNG1 gene leads to at least a 3-fold increased frequency of mutations in mitochondrial DNA (mtDNA) compared with the wild-type in *Saccharomyces cerevisiae* (53). Deciphering of the mutational spectra and mutational signature of redox stress in ssDNA of budding yeast and the signature of aging in human mitochondrial DNA indicates that the predominance of C to T substitutions is a common feature of both signatures (54). APOBEC3s deaminase-related mtDNA mutations are present in many types of cancers and often correlated with a more malignant phenotype (55–57). The mtDNA mutations impair aerobic metabolism and facilitate aerobic glycolysis. Thus, the metabolic microenvironment of chronic inflammation and cancer are similar, both of which are hypoxic and have elevated levels of lactate and low levels of nutrients. Down-regulation of UNG1 by “bad” inflammation might increase AID/APOBEC-caused mtDNA mutations, impact mitochondrial function, thus affecting energy generation from oxidative phosphorylation to aerobic glycolysis, facilitating cancer evo-dev. Thus, inflammation can select the mutated cells with metabolic adaptation.

Innate and adaptive anti-viral responses also select for malignant cells. During the chronic infection of HBV, APOBEC3B is stimulated and reduces the occupancy of H3K27me3 on the promoter of CC-chemokine ligand 2 (CCL2). By this mechanism, APOBEC3B upregulates the CCL2 to enhance the recruitment of tumor-associated macrophages (TAMs) and MDSCs. TAMs and MDSCs suppress the function of CD8^+^T cells and are associated with a poor prognosis of HCC (58). HBV can be transmitted to NK cells through exosomes, thereby inducing NK cell dysfunction and promoting HCC evolution (59, 60). The glucose metabolism of T cells is reprogramed by HBV, which leads to increased lactate production and decreased migration of T cells (61). During chronic infection, HBV promotes the recruitment of Tregs *via* activating growth factor-β (TGF-β)/miR-34a/CC-motif chemokine ligand 22 (CCL22) axis (62). Thus, chronic HBV infection might promote carcinogenesis *via* creating a cancer supportive niche.

### 2.2 The Evolutionary Characteristics of Carcinogenesis

#### 2.2.1 Genomics Evidence for Cancer Evolution

The continuous generation, selection, and accumulation of somatic mutations and cellular retrodifferentiation are two basic mechanisms underlying *Cancer Evo-Dev*. While the spontaneous rate of somatic mutations is not high enough to trigger the evolution process, many mutagenesis mechanisms can increase mutation rate in cancer genomes, such as defective DNA repair capacity, exogenous or endogenous mutagen exposures, and intrinsic slight errors of the DNA replication machinery. Mutation pattern in cancer genomes can be characterized by mutation signatures that are often linked to specific mutagenic processes, making it possible to infer which mutagenic processes have been active in patients (7). Interestingly, the cytidine deaminase-induced mutation signature is dominant in most cancers, suggesting the inflammatory immune response is the common mechanism for generating mutations (8). Among the members of APOBEC3s, APOBEC3B was identified as the major subtype responsible for the APOBEC-signature somatic mutations in multiple cancers (63).

Somatic mutations can be classified according to their effects on cancer evolution. A small proportion of the mutations can lead to aggressive phenotypes that are positively selected during the evolution process and promote cancer development. These mutations are called “driver” mutations. The driver somatic mutations affect multiple functions, like signaling pathways, EMT, and energy metabolism. The remaining mutations are “passengers” that contribute very little to carcinogenesis (64). Driver mutations are selected out at certain phases during cancer evolutionary process but may not be detectable at all stages. Mutations at advanced stage that promote the fitness of cells may replace initial driver mutations to become the dominant ones. In lung cancer patients who keep exposed to cigarettes, signatures of initial mutations (smoke-related) showed a relative decrease over time, accompanied by an increase of APOBECs-related mutations (65). Therefore, tracing the positive selection of drivers and patterns of cancer genome alteration can help demonstrate the lineage of malignant clones and the major mutagenic factors.

#### 2.2.2 Retro-Differentiation and Backward Evolution

**Figure 3:**
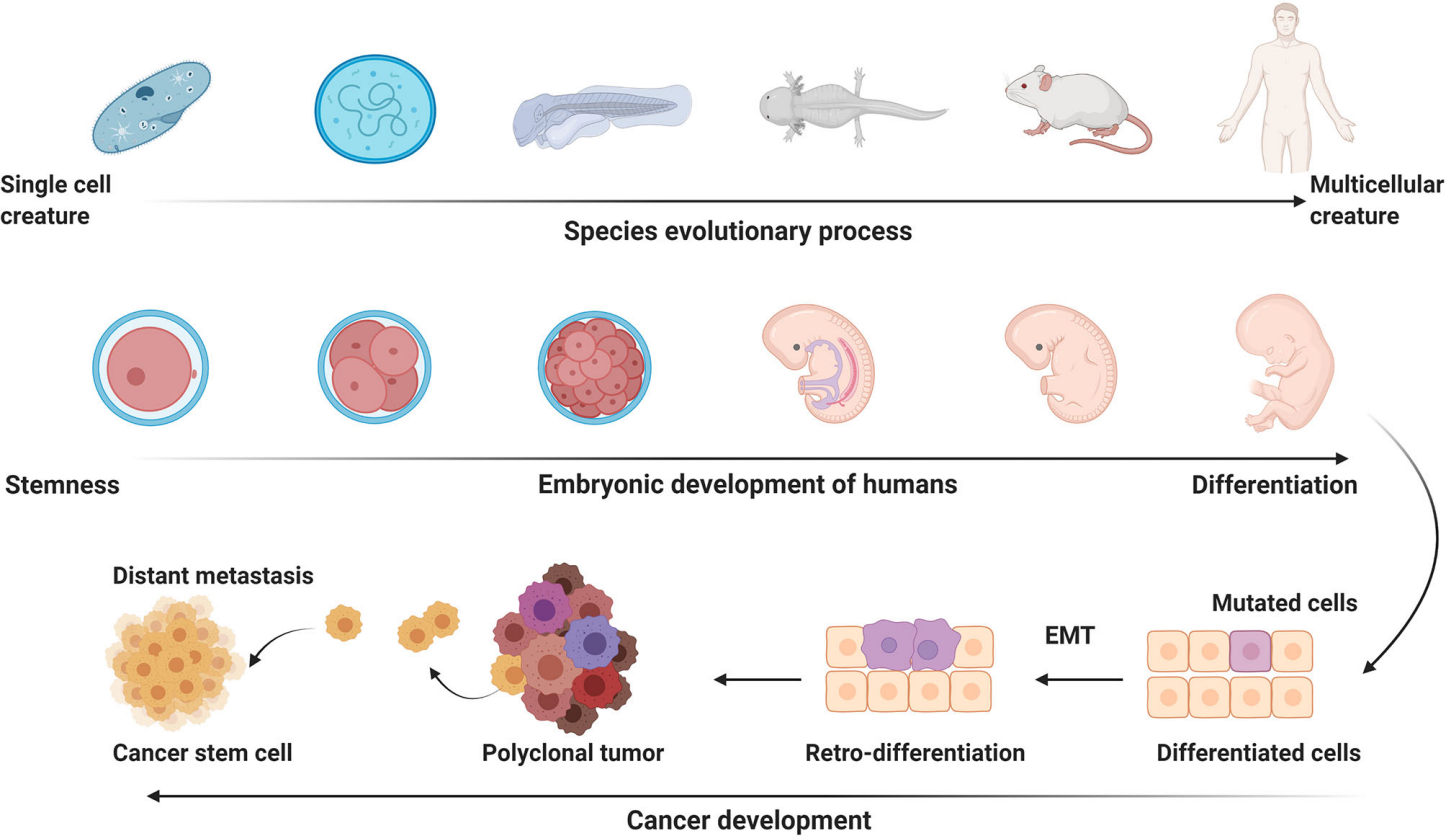
The potential link of carcinogenesis to evolution and development. Embryonic development is the process that a fertilized egg differentiates into various functional and/or structural cells to form different organs and tissues. Embryonic developmental process resembles the process of long-term animal evolution. Carcinogenesis presents a process of reverse evolution and retro-differentiation. EMT, epithelial-mesenchymal transition. This figure is created with BioRender.com.

Cancer evolution usually accompanies with retro-differentiation, a process representing reverse development (**Figure 3**). Development is referred to the process that a fertilized egg develops into an individual. This process resembles the process of long-term organic evolution morphologically, from single cell creatures to multicellular creatures and from aquatic organisms to terrestrial organisms. Some evolutionarily conserved molecules, like Hox, Hedgehog, and Myc, are essential for the developmental process, suggesting animal evolution and embryonic development have inherent mechanisms (66–69). The integration of evolution and developmental biology was termed Evo-Devo (70, 71). Carcinogenesis is characterized as a reverse-developmental process, that is, reversely develops from differentiated cells into undifferentiated cells. The embryonic factors, which are silenced after birth, are re-expressed during the process of carcinogenesis. APOBECs can promote gene demethylation and remove epigenetic memory to stabilize the pluripotent state in embryonic stem cells *via* deaminating 5-methylcytosine (5mC) or 5-hydroxymethylcytosine (5hmC) (72, 73). EMT is a landmark event of the reverse-developmental process, which is driven by transcription factors, like zinc finger E-box binding homeobox 1 (ZEB1), ZEB2, snail family transcriptional repressor 1 (SNAI1), and SNAI2. AID, a member of APOBECs family, is upregulated by inflammatory signals and induces demethylation of the promoters of ZEB1, ZEB2, SNAI1, and SNAI2. Silencing AID leads to increased methylation of CpG islands proximal to the promoters of these EMT regulators, thus inhibiting EMT and invasion of cells (74). AID-induced, CpG methylation-dependent mutagenesis is proven to be a common feature of cancer evolution (75). Therefore, it is reasonable to postulate that re-expression of embryonic factors in cancers might result from epigenetic reprogramming caused by the APOBECs family members that are usually upregulated by pro-inflammatory factors.

#### 2.2.3 Adaptation to the Inflammatory Microenvironment

To support the rapid growth of malignant cells, tumor tissues prefer to use glycolysis for energy production, even in the presence of oxygen. Glucose is more easily to be metabolized to lactate in tumor tissues than in normal tissues. This pattern of energy metabolism was identified by Otto Warburg in 1920 and was termed as Warburg effect (76). Warburg effect in TAMs promotes vascular network formation, augments extravasation of tumor cells, and induces higher levels of EMT at inflammatory foci within the tumor (77). In the microenvironment with both hypoxia and hypoglycemia, stem cell-, angiogenic-, and EMT-biomarkers as well as glycoprotein-P content and invasiveness of cancer cells are enhanced (78). The Warburg effect can provide essential energy for cell survival in the inflammatory microenvironment; furthermore, glycolysis generates the raw material for DNA synthesis of progeny cells. Thus, we believe that the Warburg effect promotes the evolutionary process of cancer under both hypoxia and hypoglycemia conditions. Infection-induced inflammation and somatic mutation are both the possible origin of Warburg phenotype. In HBV-caused HCC, the major pattern of single nucleotide variants in mtDNA is C>T, the characteristics of APOBECs-induced mutation. This kind of mutation that mainly occurs in the D-loop region of mtDNA promotes the proliferation, invasion, and metastasis of HCC cells (55). Pyruvate kinase M2 (PKM2), an alternatively spliced variant of the pyruvate kinase gene that is preferentially expressed during embryonic development and in cancer cells, alters the final rate-limiting step of glycolysis, resulting in the cancer-specific Warburg effect (79). Besides the Warburg effect, HCC cells also enhance other patterns of energy metabolism during evolution. For example, the inactivating mutation of ribosomal S6 kinase 2 (RSK2) can support cholesterol metabolism in HCC (80). Mutated cells must adapt to the inflammatory microenvironment and obtain the growth advantage in the “struggle for existence”.

#### 2.2.4 “Dead-End” Evolution

HBV belongs to the Hepadnaviridae family and is evolutionarily conservative in long-term evolution of the species (81). During HBV-induced hepatocarcinogenesis, viruses also experience the process of evolution. Two major mechanisms are responsible for the generation of HBV mutations. The first pattern is spontaneous mutations. The second viral mutation pattern is induced by host cytidine deaminases (9). The mutation rate caused by cytidine deaminases differs among different regions of the HBV genome in different host cells. APOBEC3G increases the mutation rate in the region 1 (nt. 1–1630) of the HBV genome. Endogenous cytidine deaminases edit 10 to 25% of relaxed circular double-stranded HBV DNA genomes (82). The APOBEC family has a dual effect on HBV: reduction of HBV genome copy number and induction of HBV mutations (83). The expression levels of APOBEC3s are positively correlated with the quasispecies complexity of HBV (84). The genetic polymorphisms predisposing the IL-6 induced APOBEC3B-UNG imbalance significantly promote the generation of HCC-related HBV mutations (10). Although most HBV mutations occur randomly, the direction of HBV evolution is selected under the selective pressures of chronic inflammation. In the immune tolerance phase of chronic infection, the immune pressure is weak, and most of the individual viruses are of the wild-type. Immune pressure increases with the progression of chronic inflammation, which facilitates the gradual occurrence of viral mutations, especially after HBeAg seroconversion (85, 86). HCC-related HBV mutations are selected by the immune microenvironment before the occurrence of HCC. The deficiency of CD8^+^ T cell epitopes is one of the main features of HBV mutations, which facilitates evading immune eradication (87, 88). HBV mutations posing a significant HCC risk are located mainly within the Enhancer II (EnhII)/basal core promoter (BCP)/precore region and preS regions (89–92). During the HBV-induced carcinogenic “trilogy” (chronic hepatitis, liver cirrhosis, and HCC), the species and frequencies of HBV mutations often accumulate consecutively and can be applied to predict the occurrence and development of liver cirrhosis and HCC (89, 93). SNPs of the inflammatory signal pathway genes including HLA-DP, HLA-DR, HLA-DQ, STAT3, and NF-κB have been demonstrated to maintain the chronic HBV infection and to facilitate the selection of these HCC-promoting HBV mutations (22–26). However, those viral mutants that affect the pre-cancer hepatocytes are less infectious to normal liver cells. HBV acquired during infancy or early childhood, or at the early infection stage in adults, is usually of wild-type (11, 21, 22). During the chronic infection, especially after HBeAg seroconversion, mutant HBV subgroups gradually increase. Although the HCC-related HBV mutants are present in umbilical cord blood, neonatal HBV infection is usually caused by wild-type HBV rather than by mutant subgroups. At 1–15 years in HBV-infected children, the frequencies of HCC-related mutations increase with increasing age. However, compared to their mothers who have been exposed to chronic infection for at least 25 years, the children have fewer HCC-related HBV mutations. The HCC-promoting HBV mutations increase in their frequencies with increasing age, promote the development of HCC, and are finally eradicated after its hosts died of HCC. These HBV mutants have much less opportunity to continue their evolutionary process in different individuals. Thus, HCC-promoting HBV evolution belongs to “dead-end” evolution (11, 27). In individuals with chronic HBV infection, HBV is synthesized in hepatocytes and released into the circulation at a pace of up to 10^11^ viral particles daily (94). The immune microenvironment of circulation, tumor tissue, and tumor-adjacent liver tissue determined the pace of HBV evolution. Interestingly, HBV evolves more advanced in the sera than in the tumors of HCC patients. The evolutionary similarity between the sera-derived HBV strains and adjacent tissue-derived ones is significantly stronger than that between sera-derived HBV strains and tumor-derived ones (84). Although tumor-adjacent tissues are pathologically categorized as “normal,” they are typical precancerous lesions and have already entered the middle stage of the cancer evolutionary process. The HCCs that relapse more than 2 years after resection are considered to be recurrent HCC and not a result of the initial HCC cell diffusion into the liver remnants (95, 96). The species and frequencies of certain HBV mutations in adjacent tissues are distinct in the different populations. Together with immune markers and expression levels of inflammatory genes, they can be applied to predict the prognosis of HCC patients receiving curative surgery. For example, HBV mutations in the EnhII/BCP/PreC region, such as A1762T/G1764A, can serve as predictive markers for survival and recurrence (84), indicating that HBV continues to evolve in the liver remnants until the patient dies. Antiviral therapy can block HBV evolution in adjacent tissues by easing inflammation and notably prolongs survival in HCC patients (95). Taken together, the Hepadnaviridae family members are highly conservative across species. Wild-type HBV has the advantage of infecting hepatocytes, facilitating viral spread from one individual to another, and contributing to the maintenance of its viral species. The HCC-related mutants can cause malignant transformation but have lost the advantage of person-to-person infection. Those mutants are therefore usually eliminated when their carriers pass away, which is termed “dead-end” evolution.

## 3 Discussion

### 3.1 The Role of *Cancer Evo-Dev* on Guiding Molecular Typing

Cancer is a complex disease characterized by significant heterogenicity. The accurate subtype classification is the basis for the selection of a specific therapeutic strategy (97). Significant differences exist in the outcomes of cancer patients with the same histological classification, which highlights the heterogeneity of molecular types among the same histological cancers. From the view of *Cancer Evo-Dev*, the malignant phenotypes are the outcome of the accumulation and alteration of driver mutations. As HCC has many etiological causes and experience a long evolutionary process, the somatic mutation spectrum is heterogeneous (8, 98). For example, the mutation in the gene of telomerase reverse transcriptase (TERT) is universal in HCC patients, but its mutation site and patterns are significantly different between HCC patients caused by chronic HBV infection and those caused by alcohol or HCV (99–101). The high-frequent single-gene mutations in HCV- or alcohol-induced HCC are rarely detected in HBV-induced HCC, suggesting the different mechanisms of cancer evolution. The spectrums and frequencies of altered genes vary greatly among individuals, which limits the clinical application of single-gene mutations. The mutated genes repeatedly detected in different studies are usually clustered to pathways closely related to stem-ness and embryonic characteristics. In HBV-induced HCC, the somatic mutation in HCC evolution mainly alters seven cancer-related pathways: signaling pathway related with telomere maintenance, Wnt/β-catenin pathway, P53 and cell cycle pathway, oxidative stress pathway, epigenome modifiers, RAS/RAF/mitogen-activated protein kinase pathway, and phosphatidylinositol 3-kinase (PI3K)/AKT/mTOR pathways (102). The frequencies of mutation in a single gene range from 5% to 20%. Mutation rates of the all genes in Wnt/β-catenin, p53/cell cycle control, and PI3K/mTOR pathways range from 12% to 72%. Therefore, it is promising to use combo somatic mutations as predictive and prognostic biomarkers just like gene signatures (103). Similarly, the expression signature of the signaling pathway related to stemness, gene signature, and lncRNA-miRNA-mRNA network are also applied for promoting the classification of cancer patients (104–106). Thus, driver mutations usually alter the biological behaviors of mutated cells *via* affecting functional signaling pathways and networks, while the cells with these pathways and networks are usually selected in the pro-inflammatory microenvironments.

### 3.2 The Role of *Cancer Evo-Dev* on Cancer Prophylaxis

The theory of *cancer evo-dev* can be applied in specific prophylaxis of cancer occurrence *via* reducing systemic and local chronic low-grade smoldering inflammation *via* medical and public health measures including antiviral infection, regular physical activity, pollution abatement, and psychosocial environment improvement (107, 108). Chronic physical exercise, a safe mode of intervention to prevent immunosenescence and chronic low-grade inflammation, can upregulate CD16^+^ NK and CD56^+^ NK cell activity and response, CD4^+^ T cells, CD8^+^ T cells, CD3^+^ T cells, CD19^+^ B cells, lymphocyte proliferation, neutrophils, proportion of naïve CD8^+^ T cells, and leukocyte telomere length. Chronic physical exercise also downregulates CRP, IL-6, TNF-α, proportions of senescent/exhausted killer cell lectin like receptor G1 (KLRG1)^+^/CD57^+^ and KLRG1^+^/CD28^-^ T cells, percentage of senescent naïve, central memory and effector memory CD8^+^ T cells, and senescent naïve and effector memory CD4^+^ T cells, indicating that regular physical activity and frequent exercise enhance immune competency and regulation (107, 109). Thus, regular physical activity and frequent exercise may limit or delay aging of the immune system, reduce the risk of cancer occurrence and cancer death *via* decreasing chronic low-grade inflammation. Cellular senescence and telomere length shortening are two key hallmarks of the aging process. Hyperbaric oxygen therapy can significantly increase telomere length and clearance of senescent cells and induce cognitive enhancements including attention, information processing speed, and executive functions in healthy aging adults (110, 111). The possible mechanisms involve regional changes in cerebral blood flow and possible mitigation of hypoxia-related inflammation.

### 3.3 The Role of *Cancer Evo-Dev* on Identification of New Therapeutic Targets for Cancers

As AID/APOBEC3s drive *cancer evo-dev via* causing cancer-promoting somatic mutations and viral mutations, targeting AID/APOBEC3s should be an important strategy to treat cancers. The cytidine nucleoside analogue 2’-deoxyzebularine incorporated into short ssDNAs is capable of inhibiting the catalytic activity of selected APOBEC variants derived from APOBEC3A, APOBEC3B, and APOBEC3G (112). Protein kinase A (PKA) can bind to APOBEC3B physically and phosphorylates Thr214, which is completely deprived of its deaminase activity. PKA-mediated phosphorylation inhibits A3B mutagenic activity without destructing its innate immune functions (113). Cytidine deaminase also catabolizes decitabine, the pyrimidine nucleoside analog targeting DNA methyltransferase 1, within minutes. High expression of cytidine deaminase in chemo-resistant, metastatic pancreatic ductal adenocarcinoma, and chemoresistant lymphoid malignancies is one reason for decitabine treatment failure. The cytidine deaminase inhibitor tetrahydrouridine has been added to increase therapeutic efficiency of decitabine for the treatment of advanced, chemorefractory malignant diseases in clinical trials (114, 115). These pioneering studies provide clues for developing techniques to target AID/APOBEC3s for specific treatment of cancers.

Inflammation-induced mutation, stemness, and changes in metabolism are common events in the evolutionary process of different cancer types caused by factors including chronic infection and metabolic syndrome (47, 116, 117). These common issues, including somatic mutation, epigenetic modification, activation of inflammation, and signaling pathway of stemness, could be the key events at the early stage of cancer evolution. Research on these common molecular events can reveal more efficient therapeutic targets. For example, octamer-binding transcription factor-4 (OCT4), an embryonic stem cell transcription factor, is abnormally expressed in HCC, CRC, breast cancer, and glioblastoma cell lines. Epigenetic modification of OCT4 is a common event in the evolution of cancers. Tryptophan-induced OCT4 transcription inhibitor 2-(1’H-indole-3’-carbonyl)-thiazole-4-carboxylic acid methyl ester (ITE) can significantly suppress tumor formation and tumor growth in the mouse subcutaneous tumor model and *in vivo* ectopic implantation model (118–120). TERT mutation is also a common event in different cancers. Antisense oligonucleotide technology is applied to synthesize complementary base sequences at the end of telomeres, which can block the template to inhibit the synthesis of telomerase. Telomerase inhibitor GRN163L has been proven to be effective in lung cancer, breast cancer, chronic lymphoma, HCC, and other cancers (121). Blocking the key pathways that lead to immune imbalance can relieve the microenvironment of cancer evolution. Metformin, a prescribed drug for type 2 diabetes, has been reported to protect CD8^+^ tumor-infiltrating lymphocytes from apoptosis and exhaustion characterized by decreased production of IL-2, TNF-α, and interferon-γ (IFN-γ) *via* AMP-activated protein kinase (AMPK) activation. Furthermore, metformin can relieve the abnormal energy metabolism of immune cells, increase the enrichment of CD8^+^ T cells, and correct the immune imbalance, thus achieving an anti-cancer effect (122). Inflammation promotes the generation of somatic mutation and provides the metabolic and immune microenvironment for the evolution of many cancers. From the perspective of *Cancer Evo-Dev*, inhibiting inflammation should be a basic strategy for cancer prevention and treatment. Our studies proved that anti-HBV treatment can attenuate inflammation, decreased the risk of HCC occurrence, and prevent postoperative recurrence of HCC (93, 95). Long-term use of non-steroidal anti-inflammatory drugs can also reduce the occurrence and recurrence of cancers (123–126). Targeting cancer stemness- and/or EMT-related signaling pathways might contribute to the eradication of malignant diseases (127–129).

Conclusively, the process of oncogenesis follows the rule of “mutation-selection-adaptation”. Aging and exogenous factors such as viral infection can induce chronic smoldering inflammation. Genetic predisposition contributes to chronic HBV infection and the generation of inflammation-induced HBV mutation. The elimination of chronic infection can attenuate inflammation, reducing the incidence of cancer and subsequently extending effective survival. Tumor-initiating cells obtain survival advantage during the “mutation-selection–adaptation” evolutionary process by activating a “stemness” pathway and simultaneously causing evolutionary heterogeneity. Critical molecules in a functional subnetwork that maintains and promotes the cancer evolution and development process can be demonstrated using systems biology approaches. The development of high-efficiency inhibitors that will target these critical molecules and block corresponding signal pathways could be a powerful treatment strategy in advanced cancers. *Cancer Evo-Dev* provides a new insight to integrate a large number of segmental evidence of single molecular events. This theory may help investigators to identify the common fundamental issues among the process of different cancers and to further improve the accuracy of classification and efficiency of specific treatment.

## Perspectives

In past decades, many efforts have been devoted to revealing the mechanisms by which inflammation promotes carcinogenesis. Most of these studies provide segmental and fragmental evidence, while only a few try to present a theoretical hypothesis and to promote the understanding of the fundamental laws in inflammation-induced carcinogenesis. We present the theory of *Cancer Evo-Dev* originally *via* summarizing the evidence from our studies on HBV-induced hepatocarcinogenesis and then other inflammation-related carcinogenesis. *Cancer Evo-Dev* not only helps understand the mechanisms by which inflammation promotes the development of cancers, but also lays the foundation for specific prophylaxis and targeted therapy of various cancers.

## Data Availability Statement

The original contributions presented in the study are included in the article/supplementary material. Further inquiries can be directed to the corresponding author.

## Author Contributions

WL, ZL, YC, XZ, XT, and GC collected the related paper. WL and YD drafted the manuscript. GC designed the review and extensively revised the manuscript. All authors contributed to the article and approved the submitted version.

## Funding

This work was supported by grant 2015CB554006 from the National Key Basic Research Program of China, grants 91529305, 81520108021, 81673250, 81521091, and 82003538 from the National Natural Science Foundation of China, and grant 20YF1458800 from the Shanghai Yangfan Program.

## Conflict of Interest

The authors declare that the research was conducted in the absence of any commercial or financial relationships that could be construed as a potential conflict of interest.

## Publisher’s Note

All claims expressed in this article are solely those of the authors and do not necessarily represent those of their affiliated organizations, or those of the publisher, the editors and the reviewers. Any product that may be evaluated in this article, or claim that may be made by its manufacturer, is not guaranteed or endorsed by the publisher.
